# Retropubic slings are more efficient than transobturator at 10-year follow-up: a Swedish register-based study

**DOI:** 10.1007/s00192-023-05506-4

**Published:** 2023-03-30

**Authors:** Vasileios Alexandridis, Anna Lundmark Drca, Marion Ek, Marie Westergren Söderberg, Maria Andrada Hamer, Pia Teleman

**Affiliations:** 1 Department of Clinical Sciences Lund, Lund University, Lund, Sweden; 2 Department of Obstetrics and Gynecology, Jan Waldenströms gata 47, 205 02 Malmö, Sweden; 3 Department of Clinical Science and Education, Karolinska Institutet, Stockholm, Sweden

**Keywords:** Complications, Efficacy, Long-term follow-up, Mid-urethral slings, Register, Stress urinary incontinence

## Abstract

**Introduction and hypothesis:**

Long-term performance of mid-urethral slings (MUS) and potential differences between the retropubic and the transobturator technique for insertion are scarcely studied. This study aims to evaluate the efficacy and safety 10 years after surgery and compare the two main surgical techniques used.

**Methods:**

Women who underwent surgery with a MUS between 2006 and 2010 were identified using the Swedish National Quality Register of Gynecological Surgery and were invited 10 years after the operation to answer questionnaires regarding urinary incontinence and its impact on quality-of-life parameters (UDI-6, IIQ-7) and impression of improvement, as well as questions regarding possible sling-related complications and reoperation.

**Results:**

The subjective cure rate reported by 2421 participating women was 63.3%. Improvement was reported by 79.2% of the participants. Women in the retropubic group reported higher cure rates, lower urgency urinary incontinence rates and lower UDI-6 scores. No difference was shown between the two methods regarding complications, reoperation due to complications or IIQ-7 scores. Persisting sling-related symptoms were reported by 17.7% of the participants, most commonly urinary retention. Mesh exposure was reported by 2.0%, reoperation because of the tape by 5.6% and repeated operation for incontinence by 6.9%, significantly more in the transobturator group (9.1% vs. 5.6%). Preoperative urinary retention was a strong predictor for impaired efficacy and safety at 10 years.

**Conclusions:**

Mid-urethral slings demonstrate good results for the treatment of stress urinary incontinence and tolerable complication profiles in a 10-year perspective. The retropubic approach displays higher efficacy than the transobturator, with no difference regarding safety.

## Introduction

Mid-urethral slings (MUS) revolutionized the surgical management of stress urinary incontinence (SUI) in women and are still the gold standard against which any other option is measured. Both the retropubic (RP) and the transobturator (TO) approach have been widely used, and there is currently no clear evidence for one technique to be more effective than the other [1]. Advantages and disadvantages have been noted for both, most noticeably a higher risk for bladder injury using the RP approach and a higher risk for groin pain using the TO technique. However, evidence regarding the long-term efficacy and safety of MUS as a whole is scarce [1, 2]. This poses a problem when counseling patients wishing to undergo MUS surgery, in particular younger women, and even more when concerns are raised regarding complications related to the use of synthetic mesh [3]. Such complications are, however, relatively rare, and randomized controlled studies need a substantial number of participants to reach sufficient power to detect differences between the methods [1]. Long-term follow-up periods add to these limitations, commonly resulting in high drop-out rates. National registers offer the advantage of providing a large number of participants for long-term follow-up studies, often representative of the target population [4].

The aim of this study is to examine the long-term efficacy and safety of MUS and compare the RP and TO techniques using a large cohort of women derived from a national register. The primary objective is to determine the percentage of women reporting stress urinary incontinence at 10 years and to compare the RP with the TO technique. The secondary objective is to examine the frequency of mesh-related complications and their nature.

## Materials and methods

In this cohort study we used the Swedish National Quality Register of Gynecological Surgery (GynOp) to recruit women who underwent surgery with a MUS between 2006 and 2010. GynOp was designed as a tool to facilitate potential future research and contains a wide range of preoperative characteristics of the registered women, along with information regarding surgery, perioperative period, hospital stay and status at discharge. Postoperative follow-up information is recorded on two occasions—after 2 months and after 1 year—both with questionnaires answered by women and an evaluation of the answers by a physician. There are no data about the coverage rate of GynOp concerning incontinence surgery in Sweden during the study period, but during the following 4 years, this rate was between 85 and 90% [5].

All women who underwent surgery with a MUS during the study period were included, irrespective of the type of incontinence, possible concomitant surgery, previous prolapse surgery or incontinence surgery, or previous use of vaginal implant. Women who underwent surgery with a MUS more than once during the study period were also included, and the GynOp data from the first surgery were used. Women were excluded if an absorbable or a single-incision sling (mini-sling) was used during the index surgery. The choice of questionnaires for the 10-year follow-up was partly dictated by the questionnaires used in GynOp. The term “retention” is used throughout this manuscript for the symptom defined by ICS as the complaint of inability to empty the bladder completely, both preoperatively and postoperatively.

The mailing addresses of the eligible women were obtained through the Swedish Tax Agency, and women were subsequently contacted by letter 10 years after the index operation. In November 2020, women were invited to participate in our study answering questionnaires regarding urinary tract-related symptoms (UDI-6), the impact of incontinence on quality-of-life parameters (IIQ-7) and impression of improvement, along with questions regarding background information and possible mesh-related complications postoperatively. Sling-related complications that were recorded included urinary retention, verified vaginal tape exposure, tape erosion into the bladder or bowel, inflammation/infection of the tape, fistula, reoperation, partial or total removal of the tape, repeated operation for incontinence and persisting sling-related symptoms at 10 years. Women were asked to assess whether there was a possible relation between their symptoms and the MUS surgery. Accordingly, questions were divided into two groups; questions regarding women’s symptoms irrespective of the cause, and questions regarding symptoms that women would evaluate as sling-related. Persisting symptoms were defined as those still present at the 10-year follow-up.

Participating women could answer either by mail, using enclosed envelopes, or electronically, using an online survey platform. Women who did not respond to the first invitation received a reminder letter after 2 months. Information about the study was enclosed in the invitation letter, and answering the questionnaires was regarded as consent to participate in the study. Ethical approval for this study was obtained from the Swedish Ethical Review Authority, dnr 2019-02529.

### Statistical analysis

Descriptive and analytical statistics were employed for managing and presenting the collected data. Mean, standard deviation and range were used when analyzing continuous data, whereas median, interquartile range and frequencies were used for ordinal and nominal data. The chi-squared test and Fisher’s exact test were used for comparison between groups, when nominal data were analyzed, and the Mann–Whitney U test was used with ordinal and non-normally distributed data. Binary logistic and linear regression analysis was used to adjust the results of group comparisons for possible confounders and to identify perioperative predictors for SUI, urgency urinary incontinence (UUI), impression of improvement, urinary retention and persistent symptoms due to complications at 10 years. The potential predictors that were tested were age, body mass index (BMI), parity, the American Society of Anesthesiologists physical status classification system (ASA group), diabetes, smoking, type of incontinence, previous incontinence surgery, urinary retention, antibiotic prophylaxis, type of incontinence surgery and complications within the first year postoperatively. Results are presented using the adjusted odds ratio and 95% confidence interval. The significance level was set to 5%. Analyses were performed using IBM SPSS Statistics 28 software.

## Results

**Fig. 1:**
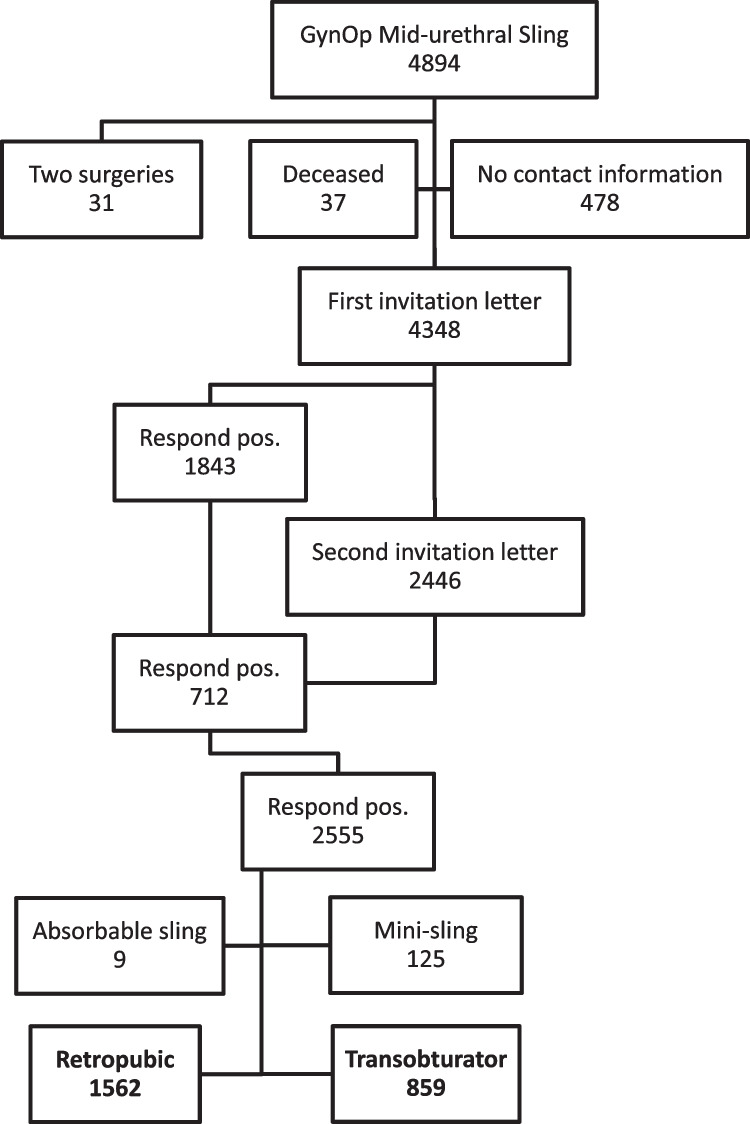
Flowchart of the study population

Out of 4894 women who were identified through GynOp as having received a MUS, 4348 were sent an invitation to participate and 2555 responded (response rate 58.8%). Examination of the participants’ operative reports from GynOp showed that nine women received an absorbable sling and 125 women a single-incision sling and were therefore excluded (Fig. 1). The remaining 2421 women who responded and were included in the analyses had a mean age of 64 years and a mean follow-up time of 10.9 years (range 9–14 years). At baseline, responders were significantly younger, had lower BMI, smoked less, and had lower ASA score and lower incidence of diabetes, previous incontinence surgery and mixed urinary incontinence (MUI) than non-responders. Baseline data for the participating women are presented in Table 1.

**Table 1:** Baseline characteristics *n* = 2421

		Retropubic	Transobturator
		*n* = 1562	*n* = 859
Age (years), mean ± SD (range)	53.2 ± 11.0 (23–85)	52.9 ± 11.1 (23–85)	53.6 ± 10.7 (23–83)
BMI, median (IQR)	25.4 (5.5)	25.2 (5.4)	25.8 (5.3)
Parity, median (IQR)	2 (1)	2 (1)	2 (1)
Smoking status, *n* (%)			
- Yes	235 (11.6)	149 (11.3)	86 (12.3)
- No	1785 (88.4)	1174 (88.7)	611 (87.7)
ASA class, *n* (%)			
- 1/2	2339 (98.6)	1512 (98.6)	827 (98.6)
- 3/4	34 (1.4)	22 (1.4)	12 (1.4)
Previous incontinence surgery, *n* (%)			
- Yes	116 (4.9)	69 (4.5)	47 (5.6)
- No	2247 (95.1)	1460 (95.5)	787 (94.4)
Diabetes, *n* (%)			
- Yes	70 (3.6)	39 (3.0)	31 (4.7)
- No	1884 (96.4)	1252 (97.0)	632 (95.3)
Type of incontinence, *n* (%)			
- SUI	1806 (76.4)	1186 (77.5)	620 (74.3)
- MUI	532 (22.5)	327 (21.4)	205 (24.6)
- UUI	11 (0.5)	7 (0.5)	4 (0.5)
- Other	5 (0.2)	4 (0.3)	1 (0.1)
- No incontinence	11 (0.5)	7 (0.5)	4 (0.5)

Retropubic sling was used in 1562 women and TO in 859 women. Bottom-to-top approach was used in 99% of the RP slings while medial-to-lateral approach was used in 68% of the TO slings. Information about the material used was available in 74% of the participating women, and it was polypropylene in all cases. At baseline, women who had received a TO sling had significantly higher BMI, lower ASA group, and lower incidence of diabetes, previous incontinence surgery and smoking than women who received the RP sling. Most women had pure SUI, according to the physicians’ reports. Perioperative data obtained through GynOp are presented in Table 2.

**Table 2:** Perioperative data, *n* (%) *n* = 2421

		Retropubic	Transobturator
Perioperative cystoscopy			
- Yes	1543 (63.7)	1527 (97.8)	16 (1.9)
- No	878 (36.3)	35 (2.2)	843 (98.1)
Bladder perforation	52 (2.1)	50 (3.2)	2 (0.2)
Antibiotic prophylaxis			
- Yes	2019 (83.5)	1415 (90.8)	604 (70.3)
- No	399 (16.5)	144 (9.2)	255 (29.7)
Perioperative complication			
- No	2334 (96.4)	1498 (95.9)	836 (97.3)
- Mild	85 (3.5)	63 (4.0)	22 (2.6)
- Severe	2 (0.1)	1 (0.1)	1 (0.1)
Bleeding	23 (1.0)	15 (1.0)	8 (0.9)
Bladder injury	47 (1.9)	45 (2.9)	2 (0.2)
Urethra injury	2 (0.1)	1 (0.1)	1 (0.1)
Complication during hospital stay			
- No	2216 (94.0)	1444 (93.9)	772 (94.0)
- Mild	136 (5.8)	89 (5.8)	47 (5.7)
- Severe	5 (0.2)	4 (0.3)	1 (0.1)
- Complication of unknown grade	1 (0.0)	0 (0.0)	1 (0.1)
Urinary retention	34 (1.4)	18 (1.2)	16 (1.9)
Infection	6 (0.3)	3 (0.2)	3 (0.4)
Pain	22 (0.9)	10 (0.6)	12 (1.5)
Reoperation during hospital stay	20 (0.8)	17 (1.1)	3 (0.3)
Complication existing at 2 months			
- No	1994 (86.6)	1307 (87.3)	687 (85.3)
- Mild	285 (12.4)	174 (11.6)	111 (13.8)
- Severe	23 (1.0)	16 (1.1)	7 (0.9)
Urinary retention	65 (2.8)	38 (2.5)	27 (3.3)
Pain	133 (5.7)	81 (5.3)	52 (6.4)
Infection	160 (7.6)	105 (7.5)	55 (7.7)
Complication existing at 1 year			
- No	2075 (94.3)	1363 (95.0)	712 (93.0)
- Mild	112 (5.1)	64 (4.5)	48 (6.3)
- Severe	14 (0.6)	8 (0.6)	6 (0.8)
All complications during first year postoperatively			
- No	1811 (80.4)	1197 (81.0)	614 (79.1)
- Mild	417 (18.5)	262 (17.7)	155 (20.0)
- Severe	25 (1.1)	18 (1.2)	7 (0.9)
Any reoperation 1 year			
- Yes	35 (1.7)	23 (1.7)	12 (1.7)
- No	2037 (98.3)	1354 (98.3)	683 (98.3)

At the 10-year follow-up, SUI was reported by 36.7% of the participants. Of all women having received a MUS, 91.6% reported being better or much better after 1 year and 79.2% after 10 years compared with their preoperative condition. At the 10-year follow-up, SUI, urgency and UUI were significantly more common among women having received a TO sling than among those in the RP group (SUI: RP 33.4% vs. TO 42.8%, *p* < 0.001) (Table 3). Women in the RP group also reported significantly lower UDI-6 scores and higher levels of improvement at 10 years (RP 80.8% vs. TO 76.3%, *p* = 0.004), even though there was no significant difference between the two groups at the 1-year follow-up (RP 92.2% vs. TO 90.6%, *p* = 0.2). No difference was detected between RP and TO groups regarding IIQ-7 scores at 10 years (Table 3).

**Table 3:** Bladder function at 10 years, *n* (%)

		Retropubic	Transobturator	*P*
Stress urinary incontinence				< 0.001*
- Yes	871 (36.7)	513 (33.4)	358 (42.8)	
- No	1501 (63.3)	1022 (66.6)	479 (57.2)	
Urgency				0.001*
- Yes	1139 (47.6)	697 (45.1)	442 (52.1)	
- No	1254 (52.4)	847 (54.9)	407 (47.9)	
Urgency urinary incontinence				0.004*
- Yes	1376 (57.8)	854 (55.6)	522 (61.7)	
- No	1005 (42.2)	681 (44.4)	324 (38.3)	
Small amounts of leakage				< 0.001*
- Yes	1216 (51.8)	742 (49.1)	474 (56.6)	
- No	1133 (48.2)	770 (50.9)	363 (43.4)	
Urinary retention				0.881*
- Yes	712 (29.7)	458 (29.6)	254 (29.9)	
- No	1683 (70.3)	1088 (70.4)	595 (70.1)	
Improvement				0.004**
- Much better	1306 (55.2)	876 (57.3)	430 (51.4)	
- Better	567 (24.0)	358 (23.4)	209 (25.0)	
- No change	247 (10.4)	143 (9.4)	104 (12.4)	
- Worse	133 (5.6)	81 (5.3)	52 (6.2)	
- Much worse	112 (4.7)	70 (4.6)	42 (5.0)	
UDI-6 score, median (IQR)	20.0 (38.9)	16.7 (38.9)	22.2 (44.4)	<0.001**
IIQ-7 score, median (IQR)	19.0 (42.9)	19.0 (42.9)	23.8 (47.6)	0.135**

* Chi-squared test, ** Mann–Whitney U test

bold entries indicate significance (p-value<0.05)

Sling-related symptoms at any time during the 10 years postoperatively were reported by 24.8% of the women, with no difference between the RP and TO techniques, while 17.7% reported persistent symptoms at the 10-year follow-up. The most common symptom that participants regarded as sling-related was urinary retention. No difference was recorded between women in the RP and TO groups regarding specific sling-related complications (exposure, erosion, inflammation/infection, fistula) or reoperation due to complications. Repeated surgery for incontinence after the first sling procedure was reported by 6.9% of the participating women, and significantly more in the TO group (9.1% vs. 5.6% in RP group) (Table 4). The same findings were recorded when analyses comparing RP and TO groups were adjusted for age, BMI, parity, diabetes, smoking, ASA group, type of incontinence and previous incontinence surgery using binary logistic or linear regression analysis, except for improvement at 10 years and UUI, where the difference between the two groups was not proven to be significant after adjusting for potential confounders.

**Table 4:** Questions at 10 years regarding sling procedure, *n* (%)

		Retropubic	Transobturator	*P*
Symptoms because of the tape				
- No	1749 (75.2)	1142 (75.7)	607 (74.1)	0.389*
- Bleeding or discharges	23 (1.0)	15 (1.0)	8 (1.0)	0.970*
- Feeling of tight vagina	60 (2.6)	35 (2.3)	25 (3.1)	0.283*
- Urinary retention	261 (11.2)	166 (11.0)	95 (11.6)	0.648*
- Other	348 (15.0)	110 (14.2)	70 (16.4)	0.154*
Have you had verified				
- Tape exposure vaginal	44 (2.0)	32 (2.2)	12 (1.5)	0.287*
- Tape erosion in the bladder	10 (0.4)	7 (0.5)	3 (0.4)	0.518**
- Tape erosion in the rectum	2 (0.1)	2 (0.1)	0 (0.0)	0.424**
- Inflammation/infection of the tape	9 (0.4)	8 (0.5)	1 (0.1)	0.123**
- Fistula	3 (0.1)	1 (0.1)	2 (0.3)	0.279**
- Other	46 (2.1)	24 (1.6)	22 (2.8)	0.062*
- No	2122 (94.9)	1383 (94.9)	739 (94.9)	0.954*
Reoperation because of the tape				0.245*
- Yes	131 (5.6)	79 (5.2)	52 (6.3)	
- No	2221 (94.4)	1450 (94.8)	771 (93.7)	
Parts of the tape removed				0.655*
- Yes	38 (1.7)	26 (1.8)	12 (1.5)	
- No	2196 (98.3)	1426 (98.2)	770 (98.5)	
Whole tape removed				0.777*
- Yes	22 (1.0)	15 (1.0)	7 (0.9)	
- No	2181 (99.0)	1424 (99.0)	757 (99.1)	
Persisting symptoms at present				0.015*
- Yes	281 (17.7)	162 (15.9)	119 (20.8)	
- No	1309 (82.3)	855 (84.1)	454 (79.2)	
Repeated operation for incontinence after first sling procedure				0.002*
- Yes	159 (6.9)	85 (5.6)	74 (9.1)	
- No	2160 (93.1)	1420 (94.4)	740 (90.9)	

* Chi-squared test, ** Fisher’s exact test

bold entries indicate significance (p-value<0.05)

Multivariable binary logistic regression analysis revealed that type of incontinence other than pure SUI, previous incontinence surgery and preoperative urinary retention were predictors for SUI, UUI and lack of improvement at 10-year follow-up. Preoperative urinary retention and complications during the first year after the surgery were predictors for persisting symptoms due to complications at 10 years. Older age was a predictor for UUI and lack of improvement at 10 years, while younger age was a predictor for urinary retention. Higher BMI, diabetes and complications during the first postoperative year were also found to be significant predictors for lack of improvement at 10 years. Looking separately at the two surgical techniques, we found that MUI and preoperative retention were predictors for lack of improvement only in the RP group, whereas higher BMI, diabetes and previous incontinence surgery were predictors for lack of improvement only in the TO group. No significant predictors were found for mesh exposure and no outcome was found to be associated with weight gain during the 10 postoperative years.

## Discussion

Our findings show that MUS perform adequately 10 years after the application in a population with both SUI and MUI, with a subjective cure rate of 63.3% and an improvement rate of almost 80%. The high improvement rate suggests a tolerable complication profile, which is supported by the low rate of serious complications reported by the women at 10 years. Still, 17.7% of the women reported having persisting symptoms because of a sling-related complication 10 years after the surgery. It might be false to attribute all the reported symptoms to MUS, but it would be difficult to fully detect the source of the symptoms even with a more objective methodology.

The RP approach seems to perform better than the TO approach, with a gradually increasing difference through time between the two techniques, as suggested by the difference in improvement after 1 year and after 10 years. The superiority of the RP technique is substantiated not only through the better results regarding SUI, but also through the UUI, urgency, UDI-6, the perception of improvement and the reoperation rates for incontinence, most of them also after adjusting for possible confounders. The impact of that difference on quality-of-life parameters (IIQ-7) was not proven to be significant. It is difficult to tell whether small changes in incontinence-related outcomes could significantly affect the women’s quality of life and to what extent [6]. No significant differences were seen regarding complications or reoperation due to complications. It is worth considering whether the higher risk for perioperative complications with a RP procedure as reported by several studies [1], and even as seen in our material, is something that truly affects women in the long run.

The subjective design for the follow-up stage in this study is a weakness, as the results are based upon the interpretation of the questions by the participating women. This is particularly important when assessing the estimated incidence of sling-related complications and reoperation. One of the main challenges in doing a large register-based cohort study is ensuring the quality of the information provided. Reviewing the participants’ medical records at a national level is not possible, and the use of diagnostic codes to find sling-related complications and reoperation is often unreliable [4]. At the same time, the greatest strength of our study is the large number of participants, essential for detecting rare incidents and comparing different techniques. The response rate of almost 60% was satisfying, considering the 10-year follow-up design of the study and the large coverage of GynOp among the Swedish population.

We believe that our study presents a relevant picture of the Swedish experience with MUS. The positive results concerning the performance of MUS might be overrepresented, considering the characteristics of the responders when compared with the total women contacted. It was not possible to obtain information regarding any concomitant prolapse or other surgery, but in Sweden it is common practice to perform MUS placement as a single procedure. The proportion of women having undergone urodynamic evaluation before the sling surgery is unknown, but the value of such an evaluation has not been substantiated in previous studies [7]. Our findings are in line with previous studies extending their follow-up period over 10 years [8–13]. In these studies, the subjective cure rate presents some significant heterogeneity (57–89%), but the impression of improvement is consistently around 80%, similar to our results. The superiority of RP slings in long-term settings relative to TO has been demonstrated previously [13–17] and has been reported even in reviews and meta-analyses [2, 18]. In our study, the frequency of both repeated incontinence surgery and revision surgery is higher than in previous register-based studies [10, 15, 17, 19–22]. However, the way repeated surgeries are recorded in such studies (mostly using diagnostic codes) can explain this disparity. Our findings regarding vaginal tape exposure concur with previous reports that show rates around 2% after 10 years [9, 12, 19, 20]. The incidence of retention varies among studies, depending on how retention is defined. When the symptom of incomplete bladder emptying is described, the incidence is, similarly to our study, quite high [10].

Older age, higher BMI, MUI and previous incontinence surgery were found to be predictors for lack of improvement, which is in agreement with previous studies [23–25]. Younger age at surgery was associated with retention, which can be attributed in part to the higher risk for postoperative pain in younger women [26]. Based on the results from logistic regression analysis, women with higher BMI, diabetes or previous incontinence surgery might benefit more from receiving a RP sling. On the other hand, women with MUI or preoperative retention might benefit from receiving a TO sling, which might be related to the higher risk for retention after a RP sling procedure, demonstrated in several studies [1, 18]. Houwert et al. reached the same conclusion concerning MUI and previous incontinence surgery for the two different techniques used [27].

The symptom of urinary retention seems to hold a key role not only preoperatively, with its predictive value for the efficacy and the safety of MUS, but also for being the most common sling-related complication reported 10 years after the surgery. The high manifestation of retention among postoperative complications has been shown previously [28]. However, the significance of obstruction for the performance of MUS has even been occasionally indicated as a working mechanism for the accomplishment of continence [29, 30]. This raises questions regarding the effect of a potentially or deliberately obstructing mechanism on an already—even partially—obstructed system, the cost that must be paid to achieve continence and the precautions needed to obtain desirable results. The importance of preoperative evaluation of subjective voiding difficulties is also greatly unexplored. Inquiring about such symptoms could prove to be as useful as measuring residual urinary volume or even performing urodynamic evaluation in search of signs of obstruction or detrusor underactivity.

One striking finding in our study is the relatively high percentage of the responders (12.7%) reporting not being aware of having received a sling. It seems that even after 2005 and in a Nordic country, information regarding a sling procedure for treating urinary incontinence was not adequately perceived by many women. We have no specific data for the 11 women who received a MUS and reported having no incontinence at baseline, but possible explanations are incorrect registration or prophylactic MUS application with concomitant prolapse surgery.

In conclusion, despite a small decline in efficacy, MUS present good long-term results with an acceptable complication profile, as suggested by the participants’ overall impression of improvement. The RP technique demonstrates significantly higher efficacy than the TO at 10 years, with no difference between the two techniques concerning complications or reoperation due to sling-related complications. Preoperative urinary retention is a strong predictor for impaired efficacy and safety at 10 years, while postoperative retention constitutes the most common sling-related complication.
